# Malignant Glaucoma After Immediate Primary Phacoemulsification for Acute Primary Angle Closure: A Case Report

**DOI:** 10.7759/cureus.37963

**Published:** 2023-04-21

**Authors:** Takafumi Suzuki

**Affiliations:** 1 Department of Ophthalmology, Shinseikai Toyama Hospital, Toyama, JPN; 2 Department of Ophthalmology, The University of Tokyo Hospital, Tokyo, JPN

**Keywords:** iridectomy, vitrectomy, phacoemulsification, acute primary angle closure, aqueous misdirection, malignant glaucoma

## Abstract

Malignant glaucoma is characterized by ciliary block or aqueous misdirection, shallowing of the anterior chamber with elevated intraocular pressure (IOP), resistance to treatment, and rapid progression to blindness. However, the exact pathogenic mechanism is yet to be established. Here, we report a case of malignant glaucoma caused by immediate primary phacoemulsification for acute primary angle closure (APAC). A 90-year-old woman, who had experienced right eye pain and blurred vision one day prior, had a cataract in the same eye without phacodonesis. The right eye IOP was 39 mmHg, preoperative anterior chamber depth was 1.00 mm, and the axial length was 22.31 mm. We diagnosed APAC in the right eye and performed phacoemulsification. On postoperative day one, the IOP decreased to the normal range (15 mmHg), the anterior chamber deepened, and the angle became open. However, one week after phacoemulsification, the anterior chamber and angle became shallower and closer again. We diagnosed the patient with malignant glaucoma, performed hyaloid-zonulo-iridectomy, and administered 1% atropine eye drops postoperatively. As a result, the IOP was limited to a 10 mmHg range with an open angle and deep anterior chamber. Malignant glaucoma can be caused by immediate primary phacoemulsification for APAC.

## Introduction

Malignant glaucoma, first described by von Graefe in 1869 [1], refers to shallowing of the central and peripheral anterior chambers of the eye with normal to elevated intraocular pressure (IOP) despite one or more patent iridotomies. The disease is characterized by ciliary block or aqueous misdirection, shallowing of the anterior chamber with elevated IOP, resistance to therapy, and rapid progression to blindness [1]. However, the exact pathologic mechanism has yet to be established.

Malignant glaucoma typically develops after glaucoma drainage surgery in patients with a history of angle closure glaucoma (ACG). However, it can also occur after intraocular surgery [2-6], topical miotic therapy [7], or in eyes that have not previously undergone surgery [8]. We have reported the clinical outcomes of immediate primary phacoemulsification for acute primary angle closure (APAC) [9]. Other reports have also investigated the outcomes of primary phacoemulsification for APAC [10-13].

To the best of our knowledge, there have been no reports of malignant glaucoma after immediate primary phacoemulsification for APAC. In this report, we present a case in which immediate primary phacoemulsification for APAC caused malignant glaucoma.

## Case presentation

A 90-year-old woman presented with a one-day history of right eye pain and blurred vision. She had a medical history of cataract surgery in the left eye but no intraocular surgery in the right eye. She was not using eye drops in either eye. At another hospital, she had undergone neodymium-doped yttrium aluminum garnet (Nd:YAG) laser capsulotomy for treatment of posterior capsular opacification in the left eye the day before her right eye symptoms occurred.

**Figure 1:**
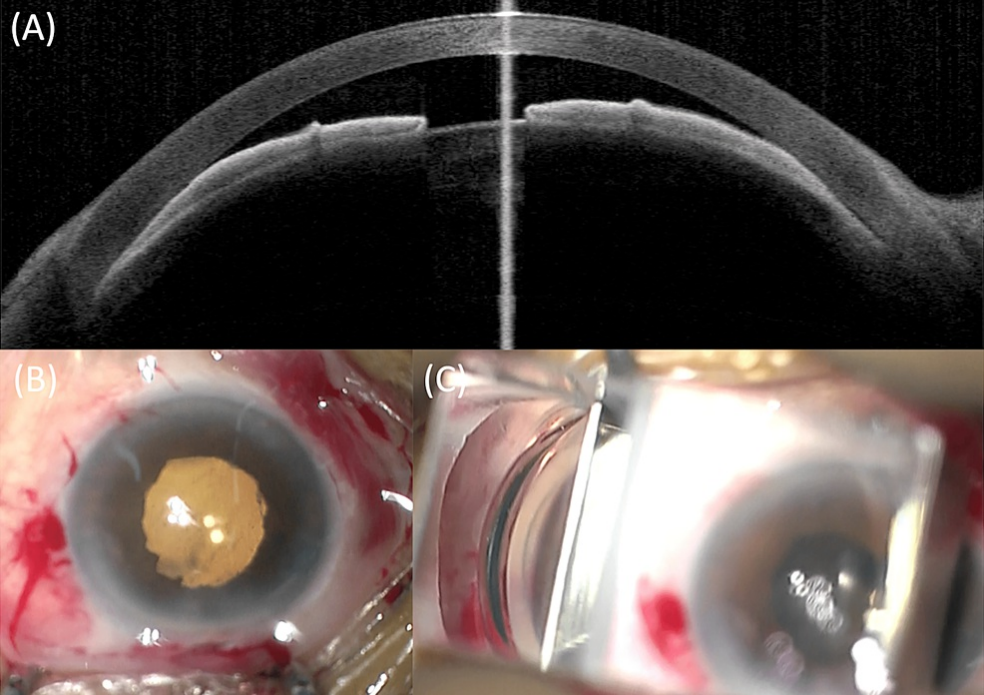
(A) Shallow anterior chamber depth and narrow angle observed at initial examination; (B) Phacoemulsification was safely performed and the intraocular lens is positioned well; (C) The angle is confirmed to be open prior to the end of surgery.

At the initial presentation at our hospital, decimal best corrected visual acuity (BCVA) was 0.1 and 1.0 in the right and left eye, respectively. She had a cataract without phacodonesis in the right eye, and an intraocular lens (IOL) in good position in the left eye. The fundus of the right eye was not visible because of corneal edema and the cataract. The IOP values were 39 mmHg and 33 mmHg for the right and left eyes, respectively. The left eye IOP was high and considered to be due to transient inflammation of the mild anterior chamber cells caused by the Nd:YAG laser capsulotomy. The right eye preoperative anterior chamber depth was 1.00 mm and the axial length was 22.31 mm (Figure 1A).

Considering the high IOP in the right eye with corneal edema, shallow anterior chamber depth, short axial length, and the sudden onset of subjective symptoms, including ocular pain and blurred vision, the patient was diagnosed with APAC. Three hours after arriving at the hospital, she underwent phacoemulsification of the right eye.

Phacoemulsification of the right eye was performed with a 2.2-mm superior corneal incision and topical anesthesia (4% lidocaine) and sub-Tenon’s anesthesia (1 mL of 2% lidocaine). After IOL insertion, the angle was observed with a Mori goniotomy lens (RE Medical Inc., Osaka, Japan) (Figures 1B, 1C). Peripheral anterior synechiae were observed at a superior angle from 11 o’clock to 1 o’clock, and goniosynechialysis (GSL) was performed with viscoelasticity only, as previously reported [9]. The angles in the other areas were open. Therefore, we performed the surgery without using a Nagata GSL needle (Inami, Japan) because the range of the open angle was considered sufficient to lower postoperative IOP.

On postoperative day one, the anterior chamber deepened and the angle became open as observed by slit-lamp examination. The IOP in the right eye decreased to 15 mmHg. Therefore, we determined that APAC was cured, and the patient was discharged. Postoperative treatment included a combination of moxifloxacin 0.5% three times daily, fluorometholone 0.1% four times daily, bromfenac 0.1% twice daily, and pilocarpine 2% three times daily to minimize the risk of peripheral anterior synechiae formation.

**Figure 2:**
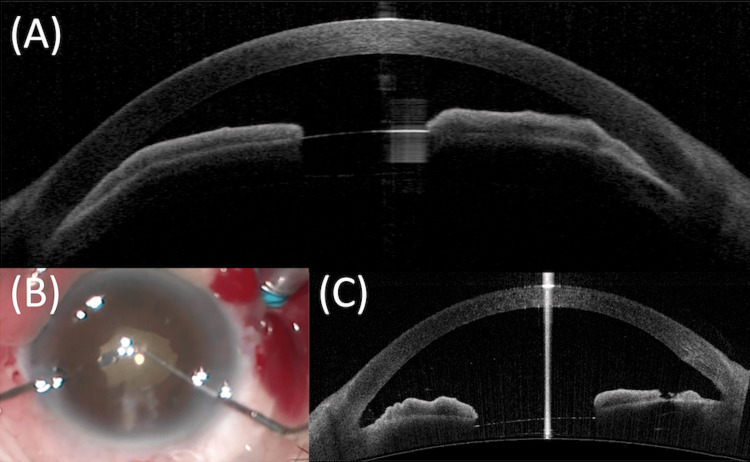
(A) Shallow anterior chamber depth and narrow angle observed again one week after phacoemulsification; (B) Peripheral iridectomy and anterior vitrectomy through the corneal tunnel, (C) The anterior chamber has deepened and the angle is open again.

One week after phacoemulsification, the anterior chamber and angle became shallower and more closed again (Figure 2A). The BCVA, anterior chamber depth, and IOP for the right eye were 0.7 (decimal), 1.37 mm, and 46 mmHg, respectively. The achieved IOL position was optimum with mild postoperative inflammation under slit-lamp examination. Based on these findings, malignant glaucoma was diagnosed. A previous report indicated the importance of establishing direct communication between the vitreous cavity and the anterior chamber for the management of malignant glaucoma [14]. Another report demonstrated the efficacy of anterior vitrectomy through iridotomy and the underlying zonule for malignant glaucoma [15]. Therefore, we performed peripheral iridectomy and anterior vitrectomy through the corneal tunnel and peripheral iridectomy (hyaloid-zonulo-iridectomy) under microscope illumination. It was performed with one port for infusion to maintain IOP with reference to the anterior vitrectomy with two ports in the previous report [5] (Figure 2B). Laser iridotomy (LI) and hyaloidotomy prior to the surgery were not performed because many problems related to LI have been reported. LI can cause corneal endothelial damage [16,17]. Between 1999 and 2001, bullous keratopathy (BK) related to LI was the second most common form of BK, accounting for 23.4% of BK cases requiring total keratoplasties in Japan [18].

On the day after the second surgery, the anterior chamber deepened and the angle became open again (Figure 2C). The BCVA, anterior chamber depth, and IOP for the right eye were 0.6 (decimal), 2.90 mm, and 21 mmHg, respectively. Therefore, we concluded that the ciliary block was completely resolved. Postoperative treatment included the administration of a combination of moxifloxacin 0.5% three times daily, fluorometholone 0.1% four times daily, and bromfenac 0.1% twice daily. Pilocarpine 2% three times daily was discontinued because it would aggravate ciliary block, the cause of malignant glaucoma [7].

**Figure 3:**
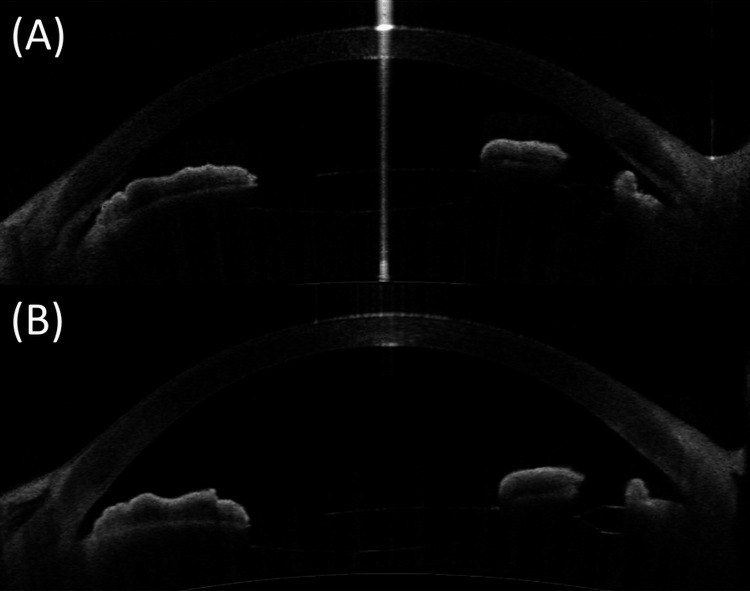
(A) The angle is narrow and the anterior chamber depth a little shallower one month after peripheral iridectomy and anterior vitrectomy; (B) The anterior chamber remains deep and the angle continues to be open with the use of atropine three months after peripheral iridectomy and anterior vitrectomy.

One month after the second surgery, the BCVA and IOP for the right eye were 1.0 (decimal) and 15 mmHg, respectively. However, the angle was narrow and the anterior chamber depth was 2.12 mm (Figure 3A). We believed that the patient had residual ciliary block. Due to her advanced age, we were hesitant to hospitalize her again for an additional vitrectomy for residual ciliary block. Therefore, we added 1% atropine once daily as an attempt to unblock (referring to a previous report [14]), which was gradually reduced. 

Three months after hyaloid-zonulo-iridectomy, the right eye BCVA, anterior chamber depth, and IOP were 1.0 (decimal), 3.00 mm, and 13 mmHg, respectively. The anterior chamber remained deep and the angle continued to be open with the use of atropine once a week (Figure 3B).

## Discussion

The most important finding in this case was that malignant glaucoma can be caused by immediate primary phacoemulsification of APAC. To the best of our knowledge, there are no other reports of malignant glaucoma after immediate primary phacoemulsification in eyes with APAC without a history of intraocular surgery. Some papers have reported malignant glaucoma after cataract surgery [3-5]. Duy et al. reported two cases of malignant glaucoma after extraocular cataract capsule extraction following a filtering procedure for chronic ACG [3]. They concluded that the previous filtering surgery and cataract surgery may have resulted in separation of the vitreous base from the pars plana, resulting in a misdirection of the aqueous humor. Reed et al. reported a case of malignant glaucoma induced by a large posterior chamber IOL [4]. Insertion of a large-diameter IOL into a small eye with an axial length of 21.7 mm without APAC is considered to cause malignant glaucoma. Al Bin Ali et al. reported 17 eyes out of 69 eyes reported in their case series developed malignant glaucoma following cataract surgery, the procedure of which was not described in detail [5]. Zhou et al. also reported that three eyes following phacoemulsification and two eyes following phacoemulsification and viscogoniosynechialysis in their case series developed malignant glaucoma [6]. Aqueous misdirection is prevalent in 0.4-6% of postsurgical cases, and it rarely occurs after uneventful phacoemulsification with in-the-bag IOL placement [14]. Hypermetropia, shallow anterior chamber, and previous angle closure or partially closed angles during surgery are predisposing factors [19,20]. The factors were considered to be associated with aqueous misdirection after phacoemulsification.

Our patient had no history of intraocular surgery and underwent immediate primary phacoemulsification for APAC. Her axial length was short, the anterior chamber was shallow, and she was at risk of malignant glaucoma, as previously reported [19,20]. The combination of hyaloid-zonulo-iridectomy, discontinuation of miotic eye drops, and administration of atropine was effective in high IOP and narrow angle after phacoemulsification, which was consistent with the diagnosis of malignant glaucoma [14]. 

We speculated that immediate phacoemulsification for APAC might have altered the direction of aqueous humor flow and caused ciliary block, similar to filtering surgery [3]. Topical pilocarpine administration after phacoemulsification might be one of the causes of malignant glaucoma. However, one month after the second surgery, the angle became narrow and the anterior chamber depth became shallow again without pilocarpine administration. Therefore, we believe that a primary cause of malignant glaucoma was immediate phacoemulsification. Further studies on these detailed mechanisms are needed.

## Conclusions

Immediate primary phacoemulsification of APAC can lead to malignant glaucoma. We can manage it through surgery and eye drop. Attention must be given to postoperative conditions such as high IOP and shallow anterior chamber depth.
